# Cerebro-Spinal Meningitis

**Published:** 1900-03-10

**Authors:** G. Hutchinson Milnes

**Affiliations:** Physician Derbyshire Royal Infirmary; Consulting Physician Derbyshire Hospital for Women.


### Hospital Clinics and Medical Progress.

### CEREBRO-SPINAL MENINGITIS.

By G. Hutchinson Milnes, M.D.Camb., Physician Derbyshire Royal Infirmary; Consulting Physician Derbyshire Hospital for Women.

In dealing with cases of so rare a disease as cerebro-spinal fever, it will, I think, be best to give first a general account of the affection, and afterwards to read the cases. Cerebro-spinal fever was first described in the early years of the century, and since then numerous epidemics have occurred in North America and the Continent of Europe, notably Southern Europe, France, and Germany. It has appeared in a partially epidemic form in Ireland, chiefly at Dublin and Belfast, and occasionally in Great Britain, at Birmingham, in Eastern country villages, and in Scotland at Kilmarnock, and a few other places. It is endemic in some parts of India. It manifests a preference for breaking out in institutions like barracks, workhouses, deaf and dumb institutes, &c. The method by which it is propagated is unknown, but it is often observed to break out unexpectedly and simultaneously at widely different places which have no connection with each other.

Since its first appearance in America in Pennsylvania in 1848, it has manifested some preference for that continent and in some of the New England towns has shown a tendency to become epidemic. At the same time isolated or sporadic cases are observed from time to time in places which have never suffered from an epidemic, and at times when no epidemic of this disease prevails in any part of the world.

Analogy would suggest that, like other infective diseases, cerebro-spinal fever has a microbic origin, and, indeed, it has already an extensive bacteriological literature.

In order to affirm without any room for doubt that any particular disease is certainly due to the presence and activity of any particular micro-organism, the following conditions must be fulfilled: (1) The parasite must be capable of being cultivated outside the body for a number of generations, and at the end of this time must possess the power of producing the disease when inoculated in a suitable manner into animals; (2) it must produce this disease, and no other; (3) the organism must be found at some period or other in every case of the disease, provided the search be properly conducted. The first condition has been fulfilled in the case of a micro-organism in connection with cerebro spinal fever; the second and third conditions have not yet been proved to be fulfilled.

Since 1882 many observations have been made public in which different micrococci were described as having been discovered in the morbid exudates of cerebro-spinal meningitis. Attempts at cultivation failed until 1886, when a bacterium, not unlike an oval coccus, was successfully cultivated from cases of this disease at Copenhagen. Inoculations of animals failed. In 1888, at Turin, cultivations were obtained which, when inoculated, produced generally sepsis, including meningitis of brain and spinal cord. At Padua, in 1890, an observer named Bonome succeeded in cultivating and inoculating a micrococcus which presented a striking resemblance in many respects to the micrococcus lanceolatus or diplococcus pneumoniæ. The micrococcus lanceolatus had already been found in the exudates of cerebro-spinal meningitis in 1886 by Vidal, in an outbreak at Orleans and Blois. Although indistinguishable in appearance from the pneumococcus, the organism found by Bonome differed from it in its behaviour in three ways: (1) It formed a peculiarly tangled growth upon agar. (2) It would not grow on blood serum, whereas the pneumococcus will grow rapidly on any but acid media. (3) Its effects on animals were different. Other observers have succeeded in cultivating from cases of cerebro-spinal meningitis a diplococcus which produces the same disease when injected into animals. A diplococcus identical in appearance with the pneumococcus is described by Dr. Osler as being characteristic of cerebro-spinal fever, and which has the peculiarity of being found in the cells of the exudates instead of in the serum or intercellular substance.

One word more before leaving the subject of bacteriology. Recent observations seem to point to the fact that these pathogenic bacteria show a tendency to be arranged in families or groups, some members of which are virulent, and others inert:—

(1) There is the typhoid group consisting, among others, of (α) Eberth's typhoid bacillus; (β) Gartner's bacillus enteritidis, the bacterium of meat poisoning; (γ) bacterium coli communis, only virulent under certain abnormal conditions.

(2) There is the diphtheria group, consisting of the genuine Klebs-Loeffler bacillus, which is the direct cause of diphtheria, and other members indistinguishable from it, some of which may be found in the mouths and noses of perfectly healthy people.

(3) The pneumonia group, which includes the diplococcus pneumoniæ, a diplococcus of exactly similar appearance to which is found in the respiratory passages in perfect health; and, finally, this diplococcus intracellularis.

We find in books on medicine that the pneumococcus is capable of causing many diseases of various organs other than pneumonia. Thus we have a pneumococcic peritonitis, a pneumococcic endocarditis, a pneumococcic meningitis of infants, and lastly this cerebro-spinal meningitis. But I would maintain that it is undesirable in the extreme to admit, except in the face of the most undeniable proof, that one micro-organism is capable of causing different diseases, and that it is better, in cases where diseases are found to be caused by organisms at present indistinguishable from each other, to assume that these organisms, although members of nearly related groups, do not all belong to the same line of ancestry.

Since bacteriology has been scientifically studied no great epidemic of this disease has occurred, so that plentiful opportunities for observation have been wanting. The present state of our knowledge is something very nearly what I have stated, but before the question is finally settled I feel confident our views will have to be considerably modified.

The morbid anatomy of cerebro-spinal fever presents the features which usually result from acute pyrexia in every organ, except in the brain and spinal cord. Here there is diffuse lympho-purulent inflammation of the pia-mater centering in the neighbourhood of the optic commissure, crura cerebri, and pons varolii at the base of the brain and on the posterior surface of the spinal cord, chiefly in the dorsal region, and lumbar enlargement. There is inflammatory softening of the cerebral cortex, and around the ventricles, which are usually distended with turbid fluid. In one case which I examined many years ago the brain was riddled with small pyæmic abscesses about the size of a large pea.

The symptoms of cerebro spinal meningitis present a great variety, and no description can possibly do justice to the many disguises it may assume.

The following are most frequently noted at the beginning: (1) Sudden onset, usually accompanied with rigor or vomiting, and nearly always headache. (2) Some affection of the central nervous system, such as delirium, lethargy, hyperæsthesia, or even coma. (3) Temperature not inordinately high, but accompanied by a slow, full, bounding pulse. To the combination of these three factors in the picture I would draw particular importance, as their recognition is most important to the formation of an early diagnosis.

*(To be continued.)*

